# The prognostic value of gender in gastric gastrointestinal stromal tumors: a propensity score matching analysis

**DOI:** 10.1186/s13293-020-00321-8

**Published:** 2020-07-23

**Authors:** Jianfang Rong, Sihai Chen, Conghua Song, Huan Wang, Qiaoyun Zhao, Rulin Zhao, Yajing He, Lili Yan, Yanping Song, Fangfei Wang, Yong Xie

**Affiliations:** 1 Department of Gastroenterology, The First Affiliated Hospital of Nanchang University, No. 17 Yongwaizheng Street, Nanchang, Jiangxi Province China; 2 Gastroenterology Institute of Jiangxi Province, Nanchang, Jiangxi Province China; 3 Key Laboratory of Digestive Diseases of Jiangxi Province, Nanchang, Jiangxi Province China; 4 Department of Gastroenterology, Affiliated Hospital of Putian University, Putian, Fujian Province China; 5 School of Pharmacy, Nanchang University, Nanchang, China

**Keywords:** Gender, Gastric gastrointestinal stromal tumor, Propensity score matching

## Abstract

**Background:**

Gastrointestinal stromal tumors (GISTs) of the stomach are the most common GISTs. The risk, incidence, and outcome of cancer are different between the sexes. Whether gender is related to the prognosis of gastric stromal tumors is unclear. Therefore, this study aims to explore the relationship between gender and gastric GIST prognosis.

**Methods:**

Data from gastric GIST patients were collected from the Surveillance, Epidemiology, and End Results (SEER) database. Propensity score matching (PSM) was performed to reduce confounding factors, and the clinicopathological features and prognosis of GIST patients were comprehensively evaluated.

**Results:**

There were 512 male patients and 538 female patients with gastric GIST. The gender of gastric GIST patients was associated with marital status, surgical treatment, tumor size, and mitotic index (*P* < 0.05). The Kaplan-Meier analysis and log-rank test revealed that male patients had a higher mortality rate than female patients (*P* = 0.0024). After matching all the potential confounding factors, the survival of the female gastric GIST patients was better than that of the male gastric GIST patients (*P* = 0.042). Cox regression analysis revealed that gender was an independent risk factor for overall survival. The risk of death was higher for males than for females (HR 1.677, 95% CI 1.150–2.444, *P* = 0.007).

**Conclusion:**

Gender could be a prognostic factor for gastric GIST survival, and male patients had a higher risk of death.

## Introduction

Gastrointestinal stromal tumors (GISTs) are the most common gastrointestinal mesothelioma and mainly caused by interstitial cells of Cajal [1]. GISTs mainly occur in the digestive tract, accounting for 1–2% of all gastrointestinal tumors in the USA. GISTs are most common in the stomach, and the incidence of gastric GISTs is 70% [2]. Approximately 30% of GISTs are malignant, and surgical resection is the main treatment for local GISTs [3]. However, some patients are still at risk of recurrence and metastasis after surgery. A population-based cohort study found that the 5-year RFS for GISTs was 70.5% [4]. The prognosis of GISTs may be influenced by many factors, including tumor size, tumor grade, and mitotic index [5].

Sexual dimorphism was associated with cancer incidence and survival [6]. Among GIST patients, men are slightly more likely to develop the disease than women (54% vs 46%) [7]. A study in Germany found that age and gender were independent risk factors for prognosis in patients with GIST, but the study only included patients in southern Germany between 2004 and 2009 [8]. However, a retrospective study did not observe any effect of gender on the prognosis of GIST [9]. Nevertheless, these studies were both retrospective studies, and the reliability of their statistical results may be weakened because other factors affecting prognosis may also vary significantly among patients of different genders. The latest data on the prognosis of gastric GISTs are still insufficient.

Therefore, this study collected gastric GIST data from 2010 to 2016 through the SEER database and used the propensity score matching (PSM) method to explore the relationship between the gender and prognosis of patients with gastric GISTs.

## Patients and methods

### Data source

The data we used was collected from the SEER database, which consists of 18 population-based cancer registries covering approximately 30% of cancer cases in the USA [10]. The SEER database collects data on patient demographics and clinical information through a population-based registry. The database is available for public cancer research and does not contain personal identifiers.

### Patient selection process

**Fig. 1:**
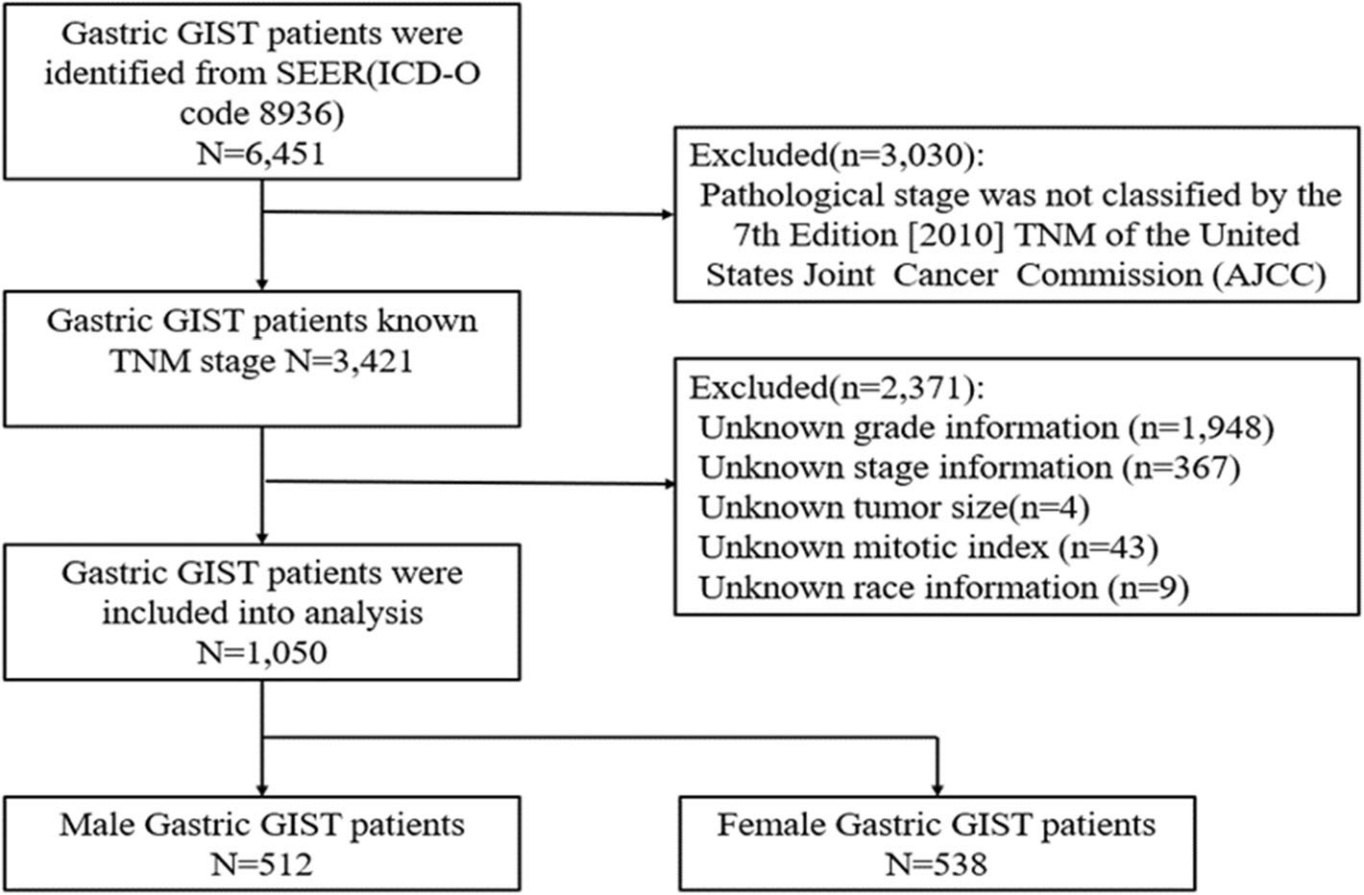
Patient selection process

Clinical information of gastric stromal tumors was extracted using the SEER*Stat software (version 8.3.2). In this study, we searched the SEER database and identified a total of 6451 gastric GIST patients from 2010 to 2016 with the diagnostic code ICD-O-8936. Patients with pathological staging that were not classified by the 7th edition [2010] TNM staging system of the American Joint Committee on Cancer (AJCC) were excluded. The exclusion criteria included the following: unknown grade information, lack of stage information, unknown tumor size, missing mitotic index information, and unknown race information. The patient selection criteria was outlined in Fig. 1.

Data on patient demographics and clinical information were obtained through the SEER database, including the following information: age, race, marital status, gender, grade, year of diagnosis, tumor size, surgical status, and mitotic index. To better analyze the data of gastric GIST patients, the patients were divided into male and female groups by gender. Race was classified as white, black, and other races; marital status was classified as married and unmarried. The tumor grade was classified into well differentiated, moderately differentiated, poorly differentiated, and undifferentiated. Gastric GIST treatment was classified into a surgical treatment group and a nonsurgical treatment group, and tumor sizes were classified as less than 2.0, 2.1 to 5.0, 5.1 to 10.0, and greater than 10.0 cm. The mitotic index was classified as less than 5, 6 to 10, and more than 10 (per 50 HPF).

### Propensity score matching

Propensity score analysis is a method to effectively adjust the confounding factors in retrospective observation studies to improve the comparability between groups [11]. To eliminate the differences in covariates between the two groups of patients, we performed propensity score matching. Propensity scores were obtained using a logistic regression model with age, race, marital status, surgical treatment, tumor grade, tumor size, and mitotic index in order to provide a one-to-one match between the male and female groups.

### Statistical analysis

The data were analyzed using SPSS Statistics version 21.0 (SPSS Inc., Chicago, IL) and R Statistical Computing Environment (R Foundation for Statistical Computing, Vienna, Austria). Continuous variables are expressed as the mean and standard deviation, and categorical data are expressed as counts and percentages for survival analysis. The baseline characteristics and group differences were compared using Pearson’s chi-square test for proportions. Survival analysis was performed using the Kaplan-Meier method (with the log-rank test) and the Cox proportional hazards model. Survival was estimated in months from the date of the diagnosis of gastric GIST to the date of death (for nonsurvivors) or the last follow-up (for survivors). *T* test was used for continuous variables. *P* value < 0.05 was considered statistically significant. All *P* values were two-tailed, and all confidence intervals (CIs) were 95% CIs.

## Results

### Clinicopathological characteristics of gastric GIST patients

As shown in Fig. 1, gastric GIST patients from 2010 to 2016 were selected from the SEER database, and there were 1050 eligible patients, including 512 male patients (48.8%) and 538 female patients (51.2%). The detailed clinicopathological characteristics of patients of different genders are shown in Table 1. Among these patients, the mean age of male patients was 64.6, and the mean age of female patients was 63.6. Most tumors are well differentiated, and a mitotic index less than 5/50 HPF accounted for 80.0%. The gender of gastric GIST patients was associated with marital status, surgical treatment, tumor size, and mitotic index (*P* < 0.05). Notably, male patients were more likely to be married (62.7%), while the female patient group had the highest proportion (49.3%) of unmarried patients. Compared with the female group, the male group was less likely to undergo surgical treatment (95.9% vs 98.1%), more likely to have large tumors (> 10.0 cm) (24.0% vs 16.4%), and more likely to have a mitotic index greater than 10/50 HPF (14.1% vs 9.7%). Age, race, and grade were similar between the two groups.

**Table 1:** Characteristics of the gastric GIST patients before and after propensity score matching

Characteristic	Before matching				After matching		
	Total, *n* (%)	Male, *n* = 512 (%)	Female, *n* = 538 (%)	*P* value	Male, *n* = 448 (%)	Female, *n* = 448 (%)	*P* value
Age (years)	1050	64.6 ± 13.0	63.6 ± 14.0	0.230	64.7 ± 13.0	64.3 ± 13.3	0.671
Race				0.858			0.501
Others	139 (13.2)	65 (12.7)	74 (13.8)		56 (12.5)	68 (15.2)	
White	651 (62.0)	321 (62.7)	330 (61.4)		285 (63.6)	274 (61.1)	
Black	260 (24.8)	126 (24.6)	134 (13.8)		107 (23.9)	106 (23.7)	
Marital status				< 0.001			0.070
Married	594 (56.6)	321 (62.7)	273 (50.7)		279 (62.3)	239 (53.3)	
Unmarried	456 (43.4)	191 (37.3)	265 (49.3)		169 (37.7)	209 (46.7)	
Grade				0.291			0.929
Well differentiated	531 (50.6)	247 (48.2)	284 (52.8)		230 (51.3)	222 (49.6)	
Moderately differentiated	315 (30.0)	154 (30.1)	161 (29.9)		137 (30.6)	140 (31.2)	
Poorly differentiated	90 (8.6)	48 (9.4)	42 (7.8)		39 (8.7)	39 (8.7)	
Undifferentiated	114 (10.8)	63 (12.3)	51 (9.5)		42 (9.4)	47 (10.5)	
Surgical treatment				0.032			1.000
No surgery	31 (3.0)	21 (4.1)	10 (1.9)		8 (1.8)	8 (2.0)	
Underwent surgery	1019 (97.0)	491 (95.9)	528 (98.1)		440 (98.2)	440 (98.0)	
Tumor size				0.001			0.145
≤ 2 cm	174 (16.6)	68 (13.3)	106 (19.7)		67 (15.0)	85 (19.0)	
2.1–5.0 cm	410 (39.0)	190 (37.1)	220 (40.9)		181 (40.4)	185 (41.3)	
5.1–10 cm	255 (24.3)	131 (25.6)	124 (23.0)		121 (27.0)	95 (21.2)	
> 10 cm	211 (20.1)	123 (24.0)	88 (16.4)		79 (17.6)	83 (18.5)	
Mitotic index/50 HPF				0.044			0.194
≤ 5	840 (80.0)	394 (77.0)	446 (82.9)		348 (77.7)	368 (82.2)	
6–10	86 (8.2)	46 (8.9)	40 (7.4)		44 (9.8)	31 (6.9)	
> 10	124 (11.8)	72 (14.1)	52 (9.7)		56 (12.5)	49 (10.9)	

**Fig. 2:**
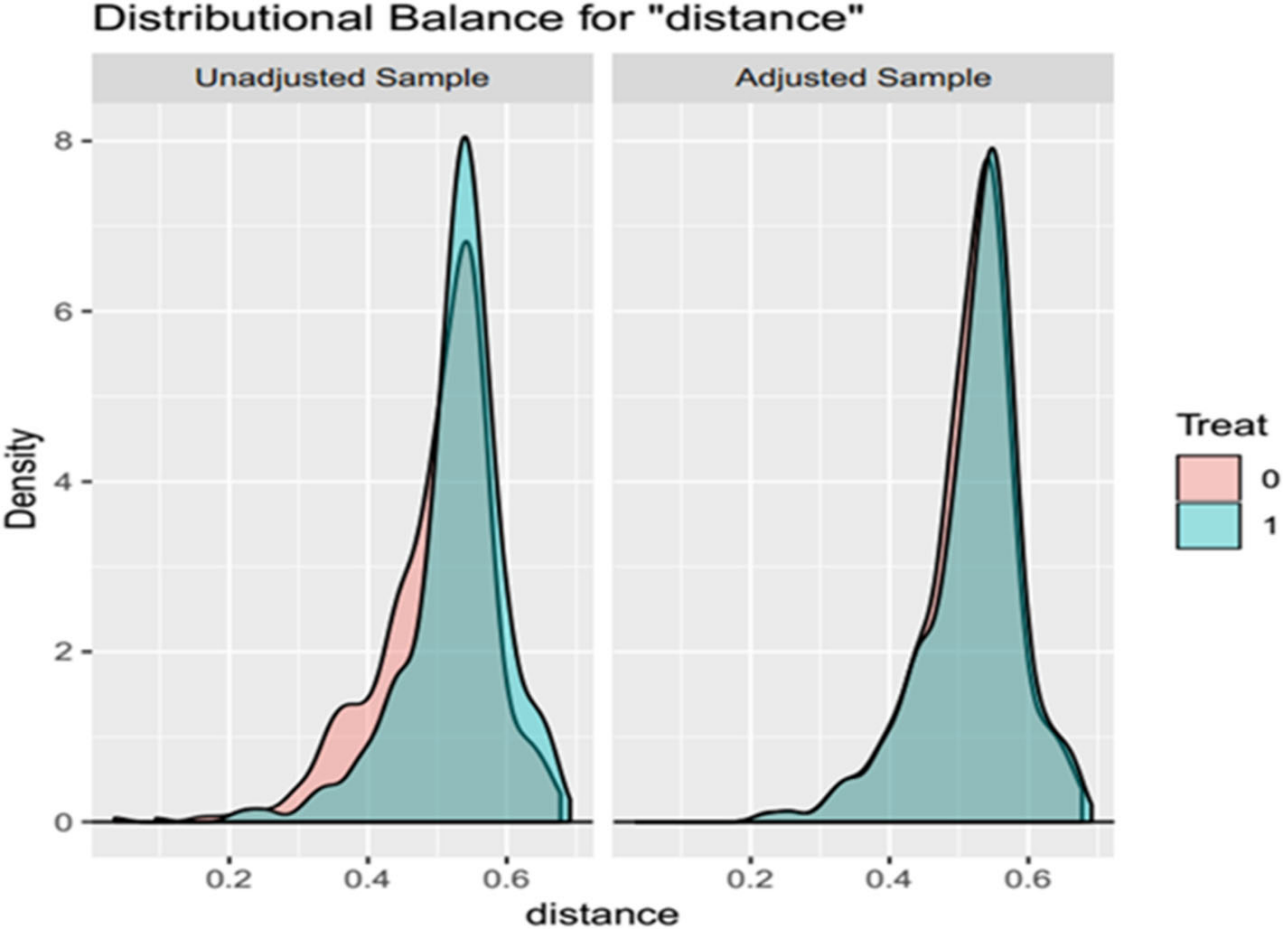
Distributional balance for “distance”

The PSM method was used to reduce the influence of confounding factors. The data of gastric GIST patients selected in the SEER database were used for propensity score matching (Fig. 2). Before propensity score matching, compared with female patients, male patients were more likely to not undergo surgery, less likely to have tumor sizes smaller than 2 cm, and more likely to have a mitotic index greater than 10/50 HPF (Table 1). However, after property score matching, all baseline variable matches between 448 male patients and 448 female patients were completely balanced (*P* > 0.05).

### Effect of gender on the overall survival of gastric GIST patients

**Fig. 3:**
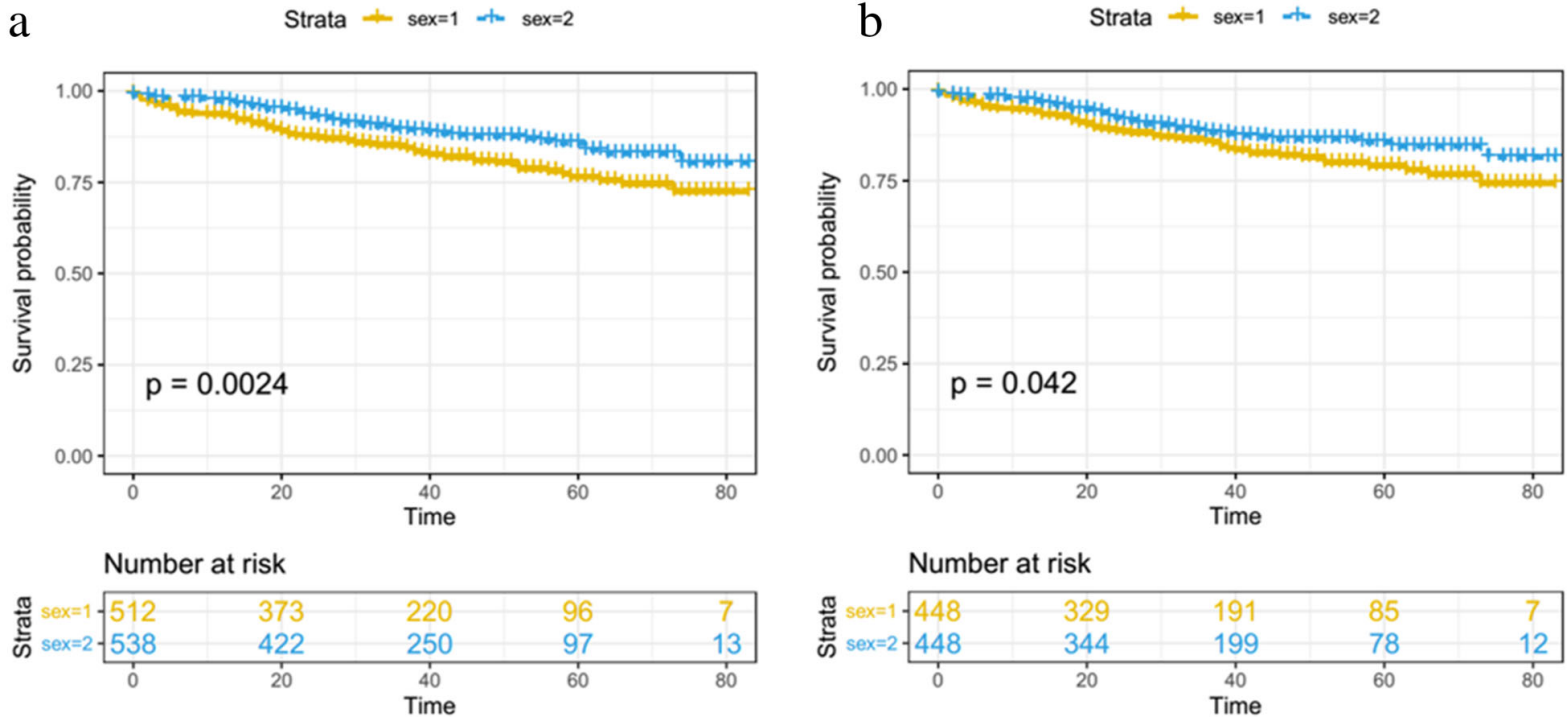
Comparison of overall survival between the male group and the female group before (**a**) and after (**b**) propensity score matching. Sex1, male; sex2, female

The Kaplan-Meier analysis and log-rank test revealed that male patients had a higher mortality rate than female patients (*P* = 0.0024) (Fig. 3a). Propensity score matching was performed to minimize any bias regarding age, race, marital status, surgical treatment, tumor grade, tumor size, and mitotic rate. After matching all the potential confounding factors, the survival of the female gastric GIST patients was still better than that of the male gastric GIST patients (*P* = 0.042) (Fig. 3b).

### Gender as an independent prognostic factor for predicting OS in gastric GIST patients

To assess the prognostic value of gender in predicting OS in patients with gastric GIST, we performed a multivariate analysis to determine its effects. The Cox proportional hazards model confirmed that gender, age, race, marital status, grade, and mitotic index were independent predictors of OS in patients with gastric GIST (Table 2). In the Cox regression model, the risk of OS was higher for males than for females (HR 1.677, 95% CI 1.150–2.444, *P* = 0.007). Gender is an independent prognostic factor for predicting OS in gastric GIST patients. Further analysis of gastric GIST data of different genders using the Cox model, we found that among male patients, the mitotic counts of 6–10/50 high power field (HPF) group had a greater risk than the < 5/50 HPF group. However, among female patients, there was no significant difference in risk between the two groups (*P* > 0.05) (Supplementary Table 1).

**Table 2:** Multivariate Cox regression analyses of prognostic factors in patients with gastric GIST after PSM

Characteristics	Hazard ratio	95% CI	*P* value
Age (years)	1.078	1.058–1.098	< 0.001
Gender			
Female [ref]			
Male	1.677	1.150–2.444	0.007
Race			
Others [ref]			0.357
White	1.212	0.653–2.247	0.543
Black	1.978	1.015–3.856	0.045
Marital status			
Married [ref]			
Unmarried	1.589	1.091–2.315	0.016
Grade			
Well differentiated [ref]			
Moderately differentiated	1.027	0.649–1.624	0.910
Poorly differentiated	1.864	1.031–3.370	0.039
Undifferentiated	2.974	1.724–5.131	< 0.001
Surgical treatment			
No surgery [ref]			
Underwent surgery	0.467	0.186–1.173	0.105
Tumor size			
≤ 2 cm [ref]			
2.1–5.0 cm	0.658	0.372–1.163	0.150
5.1–10 cm	0.879	0.483–1.599	0.673
> 10 cm	0.945	0.485–1.842	0.867
Mitotic index (/50 HPF)			
≤ 5 [ref]			
6–10	1.762	1.000–3.103	0.050
> 10	1.047	0.591–1.842	0.875

*ref* reference

## Discussion

Gastrointestinal stromal tumors (GISTs) can occur anywhere in the digestive tract but are most common in the stomach (60–70%) and small intestine (25–30%) [2, 12]. Local GISTs are mainly treated by surgery. Adjuvant treatment is not required for low-risk and intermediate-risk GISTs or high-risk GISTs that are sensitive to imatinib [13]. Approximately 30% of GISTs are malignant, and their complex biological behavior is related to the malignant transformation of the tumor, so the prognosis of patients cannot be determined by only a few factors [14]. A retrospective study did not observe the effect of gender on the prognosis of GISTs [9]. However, in this study, we found that gender is an independent predictor of gastric GIST prognosis, and male patients have a higher risk of death than female patients. To the best of our knowledge, this is the first study to study the relationship between gender and gastric GIST prognosis through PSM, and the sample size was relatively large.

This study collected data from the SEER database of 1050 gastric GIST patients. Male patients and female patients showed significant differences in marital status, surgical treatment, tumor size, and mitotic index. Compared with the female gastric GIST, male patients had the highest proportion of married, less likely to undergo surgical treatment (95.9% vs 98.1%), more likely to have large tumors (> 10.0 cm) (24.0% vs 16.4%), and have a mitotic index greater than 10/50 HPF (14.1% vs 9.7%). So, we performed a propensity score matching to avoid these confounding factors affecting the credibility of our results. At present, the prognostic assessment of GISTs is mainly based on the NIH consensus, which is based on tumor size, mitosis number, and tumor location but does not include age, gender, ethnicity, surgical treatment, or other factors [5]. Zhang et al. found that gender, tumor location, size, mitotic number, and rupture were associated with recurrence of GISTs [15]. In a previous study, Song and Tian found that marital status is a prognostic factor affecting the survival of GIST patients [16]. We found that the prognosis of gastric GIST was related to gender and marital status, which was consistent with the findings of the previous studies [16, 17].

After property score matching, all baseline variables between male patients and female patients were completely balanced, and we found that male patients had a higher risk of death than female patients. The result of our study is consistent with that of a previous study [8]. The correlation between gender differences and prognosis may be due to sex hormones. Sex hormone levels vary widely between men and women. Studies have found that sex hormones and their metabolites are involved in the development of many tumors, such as colorectal cancer, prostate cancer, breast cancer, and lung cancer [18–21]. The sex hormone signaling pathway may affect cancer susceptibility and the tumor microenvironment through a variety of mechanisms [22]. For example, fat-soluble steroid hormones act on intracellular receptors, causing their receptors to shuttle through the nucleus, affecting DNA methylation and chromatin conformation [23]. Sex hormones also regulate angiogenesis and inflammation, affecting cancer progression between the sexes. ERβ levels are usually reduced in cancer, and continuous ERβ expression is a marker of good tumor prognosis. In many cases, higher estrogen signaling activates ER, providing a protective effect from tumors [24]. Studies found that estrogen played a key role in maintaining gastrointestinal epithelial barrier function, and estrogen may reduce the risk of gastrointestinal tumors [25, 26]. In addition, a retrospective study found that GIST estrogen receptor expression was negative, but the expression of progesterone (5.4%) and androgen (17.6%) receptors was observed in some GISTs [27].

We used a Cox model to predict risk factors affecting OS in patients with gastric GIST, and we found that gender is an independent prognostic factor for gastric GIST patients. A previous study revealed that gender was associated with GI bleeding, and male GIST patients were more likely to have GI bleeding. In addition, GI bleeding is a risk factor for poor prognosis in GIST patients, men with GIST are more likely to have GI bleeding, and women have a higher OS and CSS rates [17, 28]. Currently, surgical resection is the main treatment for GISTs [3]. Even if the patient has undergone radical surgery, the recurrence rate is still 40–80% [29]. Patients with uncontrollable gastrointestinal bleeding often require emergency surgery [30]. In addition to surgical treatment, treatment with imatinib mesylate, a small molecule tyrosine kinase inhibitor, significantly improved the prognosis of GIST [31]. GIST survival rates were higher in women than in men during imatinib treatment [32]. Similarly, in a confirmed case study of c-KIT, women had higher 5-year survival rates than men (75 to 52%) [33].

Our research has some limitations. First, the data we collected from the SEER database are from American patients, so the results of the study cannot be generalized to other populations. Second, this was a retrospective study, and the SEER database lacks some treatment data, including information on targeted drug therapies such as imatinib, which is also important for survival analysis. Therefore, large, randomized, controlled studies are still needed.

### Perspectives and significance

In summary, based on the SEER database, we investigated for the first time the effect of gender on the survival of gastric GIST patients. We found that male gastric GIST patients have worse survival rates than female patients. Further research is still needed to clarify the specific mechanism of gender and gastric GIST prognosis. In addition, marital status and tumor grade are also independent risk factors that affect the prognosis of gastric GIST patients. In general, male gastric GIST patients are at greater risk, so the sooner male patients receive treatment, the better their prognosis will be. Identifying the gender differences between patients with gastric GIST is essential to increase the understanding of the more serious disease progression in male patients. In the clinical environment, doctors should pay attention to the treatment of male gastric GIST patients and strengthen their follow-up.

## Supplementary information

**Additional file 1: Table S1**. Multivariate Cox regression analyses of prognostic factors in female and male patients with gastric GIST after PSM

## Data Availability

All data are available from the corresponding author upon request.

## Abbreviations

SEER: Surveillance, Epidemiology, and End Results
GIST: Gastrointestinal stromal tumor
PSM: Propensity score matching
CI: Confidence interval
HPF: High power field
HR: Hazard ratio
OS: Overall survival

## References

[1] Rubin BP, Heinrich MC, Corless CL (2007). Gastrointestinal stromal tumour. Lancet.

[2] Miettinen M, Lasota J (2006). Gastrointestinal stromal tumors: review on morphology, molecular pathology, prognosis, and differential diagnosis. ARCH Pathol Lab Med.

[3] Roggin KK, Posner MC (2012). Modern treatment of gastric gastrointestinal stromal tumors. World J Gastroenterol.

[4] Joensuu H, Vehtari A, Riihimaki J (2012). Risk of recurrence of gastrointestinal stromal tumour after surgery: an analysis of pooled population-based cohorts. Lancet Oncol.

[5] Fletcher CD, Berman JJ, Corless C (2002). Diagnosis of gastrointestinal stromal tumors: a consensus approach. Hum Pathol.

[6] Clocchiatti A, Cora E, Zhang Y, Dotto GP (2016). Sexual dimorphism in cancer. Nat Rev Cancer.

[7] Demetri GD, von Mehren M, Antonescu CR, et al. NCCN Task Force report: update on the management of patients with gastrointestinal stromal tumors. J Natl Compr Cancer Netw 2010;8 Suppl 2:S1-S41, S42-S44. 10.6004/jnccn.2010.0116 PMC4103754 20457867

[8] Kramer K, Knippschild U, Mayer B (2015). Impact of age and gender on tumor related prognosis in gastrointestinal stromal tumors (GIST). BMC Cancer.

[9] Liu Q, Wang Y, Kong L, Kan Y (2015). Study on Clinicopathological features of gastrointestinal stromal tumor and relevant prognostic factors. Cell Biochem Biophys.

[10] Park HS, Lloyd S, Decker RH, Wilson LD, Yu JB (2012). Overview of the Surveillance, Epidemiology, and End Results database: evolution, data variables, and quality assurance. Curr Probl Cancer.

[11] Baek S, Park SH, Won E, Park YR, Kim HJ (2015). Propensity score matching: a conceptual review for radiology researchers. Korean J Radiol.

[12] Nishida T, Blay JY, Hirota S, Kitagawa Y, Kang YK (2016). The standard diagnosis, treatment, and follow-up of gastrointestinal stromal tumors based on guidelines. Gastric Cancer.

[13] Joensuu H, Eriksson M, Sundby HK (2016). Adjuvant imatinib for high-risk GI stromal tumor: analysis of a randomized trial. J Clin Oncol.

[14] Schaefer IM, Marino-Enriquez A, Fletcher JA (2017). What is new in gastrointestinal stromal tumor?. Adv Anat Pathol.

[15] Zhang X, Ning L, Hu Y, et al. Prognostic factors for primary localized gastrointestinal stromal tumors after radical resection: Shandong Gastrointestinal Surgery Study Group, Study 1201. Ann Surg Oncol. 2020. 10.1245/s10434-020-08244-9 32040699

[16] Song W, Tian C (2018). The effect of marital status on survival of patients with gastrointestinal stromal tumors: a SEER database analysis. Gastroenterol Res Pract.

[17] Wan W, Xiong Z, Zeng X (2019). The prognostic value of gastrointestinal bleeding in gastrointestinal stromal tumor: a propensity score matching analysis. Cancer Med.

[18] Graff RE, Meisner A, Ahearn TU (2016). Pre-diagnostic circulating sex hormone levels and risk of prostate cancer by ERG tumour protein expression. Br J Cancer.

[19] Lin JH, Zhang SM, Rexrode KM (2013). Association between sex hormones and colorectal cancer risk in men and women. Clin Gastroenterol Hepatol.

[20] Moore SC, Matthews CE, Ou SX, et al. Endogenous estrogens, estrogen metabolites, and breast cancer risk in postmenopausal Chinese women. J Natl Cancer Inst. 2016;108. 10.1093/jnci/djw103 PMC5858156 27193440

[21] Stapelfeld C, Neumann KT, Maser E (2017). Different inhibitory potential of sex hormones on NNK detoxification in vitro: a possible explanation for gender-specific lung cancer risk. Cancer Lett.

[22] Rubin JB, Lagas JS, Broestl L (2020). Sex differences in cancer mechanisms. Biol Sex Differ.

[23] Nugent BM, Wright CL, Shetty AC (2015). Brain feminization requires active repression of masculinization via DNA methylation. Nat Neurosci.

[24] Yu CP, Ho JY, Huang YT (2013). Estrogen inhibits renal cell carcinoma cell progression through estrogen receptor-beta activation. PLoS One.

[25] Grishina I, Fenton A, Sankaran-Walters S (2014). Gender differences, aging and hormonal status in mucosal injury and repair. Aging Dis.

[26] Nie X, Xie R, Tuo B (2018). Effects of estrogen on the gastrointestinal tract. Dig Dis Sci.

[27] Lopes LF, Bacchi CE (2009). Androgen receptor expression in gastrointestinal stromal tumor. Appl Immunohistochem Mol Morphol.

[28] Chen Z, Lin RM, Bai YK, Zhang Y (2019). Establishment and verification of prognostic nomograms for patients with gastrointestinal stromal tumors: a SEER-based study. Biomed Res Int.

[29] Iorio N, Sawaya RA, Friedenberg FK (2014). Review article: the biology, diagnosis and management of gastrointestinal stromal tumours. Aliment Pharmacol Ther.

[30] Liu Q, Li Y, Dong M, Kong F, Dong Q (2017). Gastrointestinal bleeding is an independent risk factor for poor prognosis in GIST patients. Biomed Res Int.

[31] Caram MV, Schuetze SM (2011). Advanced or metastatic gastrointestinal stromal tumors: systemic treatment options. J Surg Oncol.

[32] Chiang NJ, Chen LT, Tsai CR, Chang JS (2014). The epidemiology of gastrointestinal stromal tumors in Taiwan, 1998-2008: a nation-wide cancer registry-based study. BMC Cancer.

[33] Rubio J, Marcos-Gragera R, Ortiz MR (2007). Population-based incidence and survival of gastrointestinal stromal tumours (GIST) in Girona, Spain. Eur J Cancer.

