# Supplementary material for: The prognostic value of gender in gastric gastrointestinal stromal tumors: a propensity score matching analysis
Source: Biol Sex Differ. 2020 Jul 23;11:43. doi: 10.1186/s13293-020-00321-8 (PMC7376864; doi:10.1186/s13293-020-00321-8)
Supplement: Supplementary file 1 — Additional file 1: Table S1. Multivariate Cox regression analyses of prognostic factors in female and male patients with gastric GIST after PSM [file 13293_2020_321_MOESM1_ESM.docx]

Supplementary Table 1: Multivariate Cox regression analyses of prognostic factors in female and male patients with gastric GIST after PSM.

| Characteristics |  | Female |  |  |  | Male |  |
| --- | --- | --- | --- | --- | --- | --- | --- |
|  | Hazard Ratio | 95% CI | P value |  | Hazard Ratio | 95% CI | P value |
| Age (year) | 1.088 | 1.056-1.120 | <0.001 |  | 1.068 | 1.042-1.094 | <0.001 |
| Race |  |  |  |  |  |  |  |
| Others [ref] |  |  |  |  |  |  |  |
| White | 1.731 | 0.598-5.008 | 0.311 |  | 0.901 | 0.411-1.972 | 0.794 |
| Black | 2.078 | 0.648-6.662 | 0.218 |  | 1.798 | 0.789-4.102 | 0.163 |
| Marital status |  |  |  |  |  |  |  |
| Married [ref] |  |  |  |  |  |  |  |
| Unmarried | 1.691 | 0.870-3.285 | 0.121 |  | 1.437 | 0.876-2.357 | 0.151 |
| Grade |  |  |  |  |  |  |  |
| Well differentiated [ref] |  |  |  |  |  |  |  |
| Moderately  differentiated | 1.131 | 0.537-2.382 | 0.747 |  | 0.956 | 0.526-1.735 | 0.882 |
| Poorly differentiated | 2.051 | 0.755-5.571 | 0.159 |  | 1.926 | 0.903-4.106 | 0.090 |
| Undifferentiated | 2.684 | 1.144-6.300 | 0.023 |  | 2.578 | 1.224-5.432 | 0.013 |
| Surgical treatment |  |  |  |  |  |  |  |
| No surgery [ref] |  |  |  |  |  |  |  |
| Underwent surgery | 0.522 | 0.151-1.805 | 0.305 |  | 0.453 | 0.109-1.887 | 0.277 |
| Tumor size |  |  |  |  |  |  |  |
| ≤2 cm [ref] |  |  |  |  |  |  |  |
| 2.1-5.0 cm | 0.633 | 0.250-1.601 | 0.334 |  | 0.666 | 0.323-1.374 | 0.271 |
| 5.1-10 cm | 1.359 | 0.519-3.556 | 0.532 |  | 0.649 | 0.297-1.415 | 0.277 |
| >10 cm | 1.407 | 0.492-4.020 | 0.524 |  | 0.692 | 0.288-1.662 | 0.410 |
| Mitotic index  /50HPF |  |  |  |  |  |  |  |
| ≤5 [ref] |  |  |  |  |  |  |  |
| 6-10 | 1.245 | 0.429-3.616 | 0.687 |  | 2.325 | 1.166-4.635 | 0.017 |
| >10 | 0.621 | 0.214-1.804 | 0.381 |  | 1.379 | 0.683-2.782 | 0.370 |
